# The National Study of Daily Experiences: Protocol for Assessments of Daily Stress, Well-Being, Health, and Salivary Biomarkers in a Longitudinal Cohort

**DOI:** 10.2196/76453

**Published:** 2025-10-02

**Authors:** David M Almeida, Susan T Charles, Jennifer R Piazza, Robert S Stawski, Kelly E Cichy, Eric S Cerino, Jonathan Rush, Jody S Nicholson, Jennie C Holmberg, Natalie Cramer, Jacqueline Mogle

**Affiliations:** 1 Department of Human Development and Family Studies Pennsylvania State University University Park, PA United States; 2 Center for Healthy Aging Pennsylvania State University University Park, PA United States; 3 Department of Psychological Science University of California, Irvine Irvine, CA United States; 4 Department of Public Health California State University, Fullerton Fullerton, CA United States; 5 Department of Human Development and Family Studies Utah State University Logan, UT United States; 6 Department of Human Development and Family Science Kent State University Kent, OH United States; 7 Department of Psychological Sciences Northern Arizona University Flagstaff, AZ United States; 8 Department of Psychology University of Victoria Victoria, BC Canada; 9 Department of Psychology Clemson University Clemson, SC United States; 10 RTI Health Solutions Durham, NC United States

**Keywords:** daily diary, ecological momentary assessment, publicly available data, cortisol, stress

## Abstract

**Background:**

Modern psychology has long recognized that understanding human behavior requires knowledge about a person’s current context, which is often examined through daily diary studies. These studies offer ecologically valid insights into how everyday experiences—particularly stressors—affect health and well-being. The National Study of Daily Experiences (NSDE) addresses a critical gap by applying this approach in a large, longitudinal, and publicly accessible study that captures daily life across adulthood.

**Objective:**

The NSDE is the largest and longest-running publicly accessible daily diary study in the United States. The purpose of this paper is to provide a guide for researchers interested in initiating similar naturalistic studies and to facilitate research using the existing NSDE data.

**Methods:**

The NSDE includes 3510 adults (aged 24-97 years), yielding over 42,000 days of information to capture how daily life changes with age, over time, and across different cohorts, and how these daily experiences predict later health and well-being. This intensive longitudinal dataset includes an 8-day daily diary collected via phone survey, spans more than 20 years, and consists of 2 longitudinal datasets. During the daily phone interviews, participants provide reports of their experiences regarding daily events, including their stressors (Daily Inventory of Stressful Events), as well as their physical health indices, emotional experiences, and cognitive health. In addition, saliva is collected concurrently with days 2-5 of the daily phone interviews (4 collections per day for 4 consecutive days) and is used to measure biomarkers such as cortisol and alpha amylase.

**Results:**

Recruitment began in 1995, with data collection occurring every 9-10 years. The most recent data collection is ongoing through 2027. All NSDE data are housed under the Midlife in the United States (MIDUS) study umbrella, with archived and updated datasets made available to the public on the online portal, MIDUS Colectica.

**Conclusions:**

Results from the NSDE have refined our understanding of daily stress processes. The study’s timescale has provided insight into daily life for hundreds of studies, yet much more can be learned from using these data. Microlongitudinal measures and combinations of factors provide for new avenues of research and promise for better understanding of health and aging. Moreover, NSDE data can be combined with datasets from neuroscience, biomarker, and macrolongitudinal subprojects from MIDUS to examine health-related processes. In addition to offering information on how to use the NSDE, this protocol serves as a resource for secondary data analyses and an outline for investigators wishing to replicate an intensive assessment design to other populations and research questions to continue to refine our understanding of how daily stress processes influence health and well-being.

**International Registered Report Identifier (IRRID):**

DERR1-10.2196/76453

## Introduction

### Background

In 1995, the Midlife in the United States (MIDUS) study was launched to examine health and well-being across middle adulthood. Embedded in the MIDUS was the National Study of Daily Experiences (NSDE), an 8-day diary study that assessed the daily behaviors, thoughts, and emotions of approximately 1500 people ranging from 25 to 75 years of age. Until then, midlife had been a relatively neglected life stage in developmental, behavioral, and social science research. Researchers focused largely on ontological changes until early adulthood, followed by a relatively smaller group examining older adults. Midlife was often characterized as a period of stability before the onset of physiological declines in older adulthood. Early life risk factors in childhood for later health were widely recognized, but few researchers were examining the importance of risk and resilience factors in midlife for predicting healthy aging [1].

The primary goal of the NSDE was to investigate the contributions of behaviors, thoughts, and emotions in daily life to functional aging processes. Key to this innovation was focusing on stress in daily life [2] and how it contributed to health, well-being, and aging. Prior research on stress in adulthood focused primarily on major life events (eg, marriage, bereavement, and retirement) and their impacts on health and well-being [3]. In contrast, daily stressors (eg, minor everyday conflicts or irritations) [4,5] received much less attention but offered considerable promise for understanding contextual factors impacting life as it is lived. Embedded in the NSDE was the Daily Inventory of Stressful Events (DISE), a set of questions that capture multiple aspects of an individual’s experience that articulate stress processes [6].

The first wave of NSDE was launched with a focus on understanding how daily stressors influence daily health and well-being. The second wave of data collection, nearly 10 years later, expanded its focus on the role of daily events to include not only stressors but also daily positive events. The goals of the second data collection were to examine how data collected earlier predicted later health and to expand the study of the role of daily events (both positive and negative) to mental and physical well-being. Questions about positive events were added, as well as more questions to characterize the stressor experience. To better capture physiological mechanisms related to physical health, 4 days of saliva samples were collected with the aim of documenting diurnal patterns of cortisol, the primary glucocorticoid in humans and an ambulatory marker of stress and endocrine function. Saliva collection has many benefits in a study of this capacity, including the ability to be self-collected in the participants’ natural environment, the collection is noninvasive, salivary cortisol levels are stable allowing for shipment and assay by an off-site laboratory [7], and salivary cortisol levels correlate with blood cortisol levels [8]. In addition, a further aim of this second wave was to examine how individual characteristics (personality, socioeconomic status, and caregiving status) shaped the stress response; thus, additional participants were included to gain a larger, more heterogeneous national sample.

A few years later, a new sample of adults, called the refresher sample, was collected using this same design. The purpose of this new cohort was to supplement the existing sample and examine an additional aim. In 2008, the United States had experienced the Great Recession, the biggest economic decline since World War II. Two years later, epidemiologists and demographers noticed a disconcerting trend. Life expectancy in the United States, which had been increasing steadily since it had been first recorded in 1947, started slowing in 2010 and then declined [9,10]. A main reason for this decline was excessive deaths in the United States among adults aged 45-54 years, referred to as deaths of despair (ie, deaths from drug overdoses, suicide, and alcoholic liver disease) [11,12]. Life had become more stressful, especially for people in midlife.

The collection of a refresher cohort (beginning in 2012) allowed for examination of how the daily stress process may have varied across groups assessed before and after this event. A third wave of data from people who had participated previously (NSDE3, beginning in 2016) made it possible to examine these changes longitudinally. In addition to documenting how macrolevel stressors (eg, economic and social forces) affected stress in daily life, new questions were added about daily cognition to include not only measures of daily emotional and physical well-being but also cognitive well-being. In 2023, data collection for a fourth wave of the original study and a second wave of the refresher cohort began.

### Aim of This Study

Currently, NSDE consists of more than 42,000 daily interviews spanning over 20 years. Almost 300 papers and chapters have been published using NSDE data, with the number of papers increasing each year (see Daily Experiences Publications for Researchers [13]). These papers shed light on the contextual factors (eg, socioeconomic status) and daily experiences that shape health and well-being across the adult life span [14], but more discoveries are possible. For example, in addition to stressors, the extensive NSDE interview has allowed for examination of a broader array of individuals’ daily experiences, such as exposure to nature and well-being [15], everyday discrimination and cognition [16], and the effects of daily activity diversity on cognitive and psychological well-being [17,18].

The purpose of this paper is to describe the study’s design, the sample and measures, and analytic strategies necessary to test questions using this complex measurement burst study design. We do so with 2 aims: that people gain the knowledge necessary to use the publicly accessible NSDE (available through the MIDUS portal [19]), and that they consider initiating similar studies in the future, building our methods to further investigate how our daily lives exert lasting influences on our daily health.

## Methods

### MIDUS and NSDE Project Overview

The MIDUS study is a large, cross-project longitudinal study composed of multiple subprojects linking behavioral, psychosocial, and biological metrics to health and function across adulthood [20,21]. The NSDE is one of these embedded projects. MIDUS includes 3 waves of data beginning in 1995. The original participants have been followed at least every 10 years postbaseline (with wave 2 in 2004-05 and wave 3 in 2012-13), with new participants included in the second wave, including a city-specific subsample of Black adults, aged 35-85 years. In 2012, a “refresher sample” was recruited for new data collection and to refresh the existing pool of participants. The initial participants, as well as the subsample of Black adults, are referred to as the “core sample” and have completed up to 3 waves (with wave 4 currently in progress), whereas the refresher sample consists of a single wave (with wave 2 in progress at the time of this writing).

**Figure 1:**
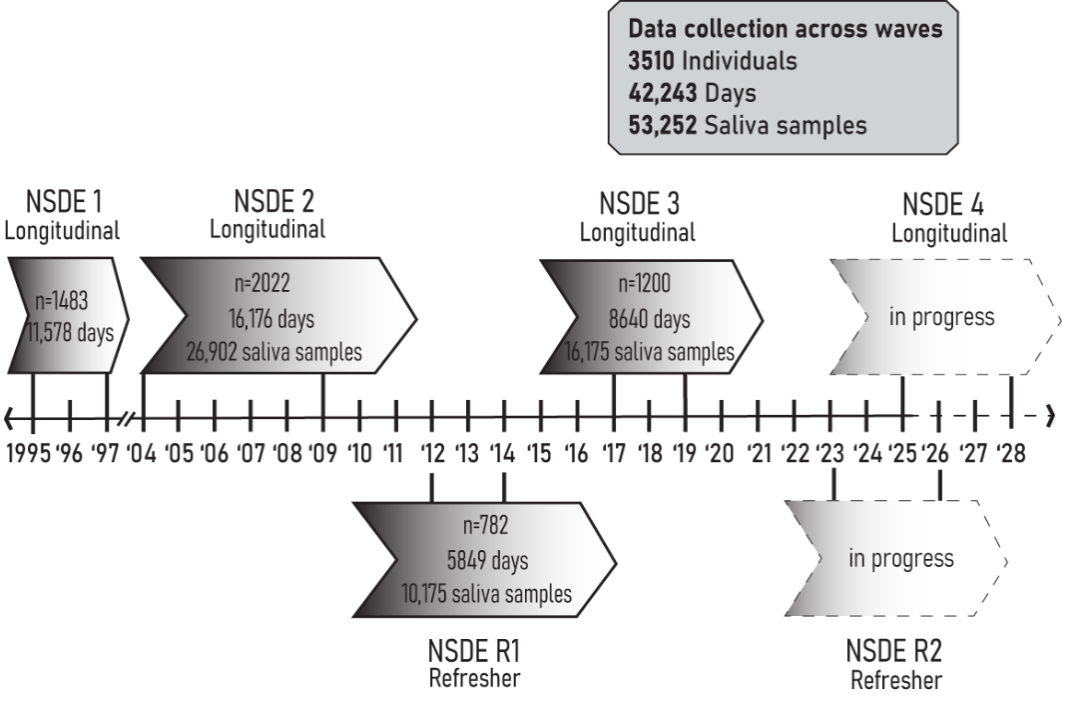
Timeline of National Study of Daily Experiences (NSDE) data collection across waves. NSDE 1-4 represent the core cohort, and NSDE R1 and R2 represent the refresher cohort. Dashed lines represent data collection in progress.

The NSDE is composed of a subset of participants from MIDUS (both the core and refresher samples) who consented to a series of daily interviews after having completed the MIDUS survey [22,23]. The NSDE collection is initiated about 3-6 months after the MIDUS Survey Project interview (see Data Collection Timeline for Researchers [13]), and home saliva sampling kits are sent in batches to 25-50 participants at a time. Figure 1 outlines the timing of waves for the NSDE, providing an overall picture of data collection for the core and refresher cohorts, including the number of participants, interview days, and saliva samples. This figure presents the timing of assessments collected via phone surveys and saliva samples, including our ongoing data collection.

**Figure 2:**
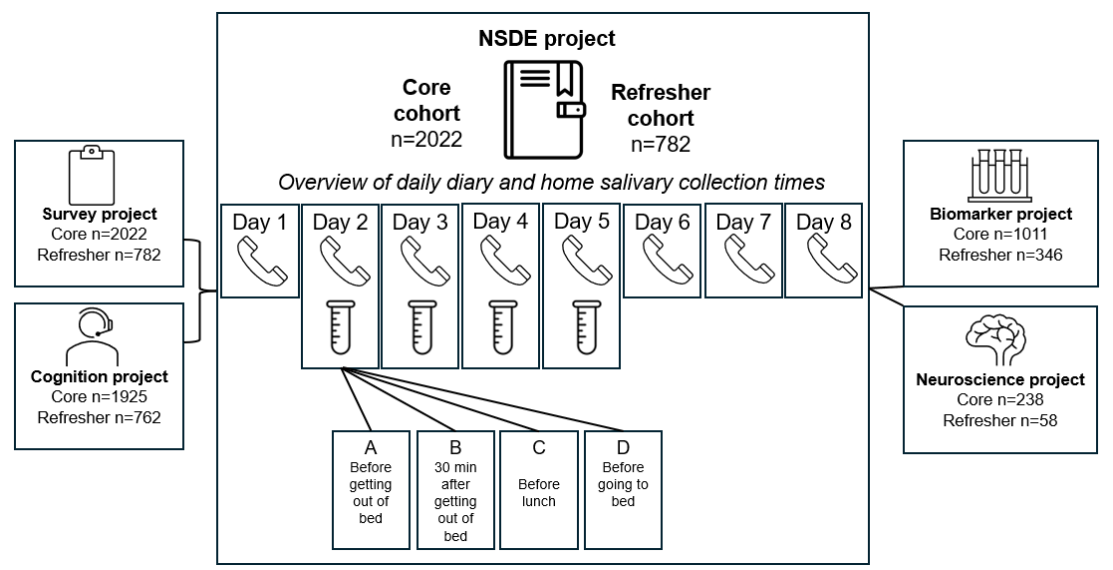
Cross-project synergy with the National Study of Daily Experiences (NSDE). N=2022 for the core cohort and N=782 for the refresher cohort reflect maximum number of respondents at NSDE 2 (core cohort) and NSDE R1 (refresher cohort), respectively.

Figure 2 illustrates a focused view of the flow and design of wave 2 data collection, providing an example of how the NSDE is linked to other projects within the larger MIDUS study. At each wave, NSDE participants complete brief evening surveys via telephone interview (8 consecutive days). On the final evening of the telephone survey, participants are asked additional questions to reflect on their week. Saliva collection was added to the protocol beginning in the second wave (4× per day for 4 consecutive days, interview days 2-5).

### NSDE Study Sites and Participants

A central MIDUS headquarters at the University of Wisconsin–Madison currently coordinates NSDE participant selection and recruitment, which began in 1995-1996. Telephone interviews were conducted from the University of Michigan and the University of Arizona (wave 1), and then the Survey Research Center at Penn State University in all following years. Saliva samples were processed at the University of Wisconsin–Madison. Participants from the NSDE initial cohort consisted of 1483 individuals aged 25-74 years and included a nationwide sample of individuals, including siblings of the sample, oversampling from US metropolitan areas, and over 200 twin pairs [20,21,23,24]. Participant details at baseline and at the longitudinal follow-up are available [25].

### Recruitment and Daily Diary Phase

Recruitment of the initial core sample of MIDUS participants used a national random-digit dialing method from working telephone banks for people in the United States. Those enrolled in wave 1 of the MIDUS study were asked to participate in additional MIDUS core projects and data collection waves. For the new refresher sample, a sampling frame consisting of all possible landline and mobile phone numbers was used. All NSDE participants selected for the 8-day daily diary protocol had previously participated in the larger MIDUS survey study. The participants were sent letters of introduction and information about requests for this study. Starting in the second wave of NSDE data collection, salivary assessment kits were included by mail. Within 1 week of sending the introductory letter and salivary kits, participants were contacted via telephone to answer any questions and enroll willing participants. The initial and final NSDE interviews last approximately 15-20 minutes, while the other 6 interviews last approximately 10-15 minutes. A computer-aided telephone interview, which enables skip patterns, open-ended probe questions, and keypunch data entry during the interview, was used for participant interviews.

### Ethical Considerations

This protocol was approved by Penn State University’s Institutional Review Board (PRAMS00042558), Clemson University’s Institutional Review Board (2023-0053), and Northern Arizona University’s Institutional Review Board (2020855). All historically collected data were deidentified before the analyses, as will future datasets currently in collection. Informed consent was obtained from all participants prior to the survey and saliva collection, and participants received the equivalent of US $25 for compensation.

### Home Data Collection: Salivary Sample Kits

NSDE home saliva collection kits were designed to maximize ease of use by participants. Each kit contained a brochure, a recruitment letter addressed to the participant, and an informed consent sheet. These items highlighted the goals of MIDUS and NSDE, communicated why participants had received the kit, and provided research staff contact information. Along with these instructional materials were the following items: 16 prelabeled salivettes in a double bag with an absorbent pad, printed inserts including collection instructions, a saliva collection sheet for recording collection times, and a daily medication use form for disclosing prescription and nonprescription medication that may interfere with the interpretation of cortisol data. All items were labeled with a unique, nonpersonally identifiable kit number. An insert was included with detailed instructions about how to schedule a package pickup upon completion of the data collection, and participants were given a toll-free number should they encounter issues. Participants were also provided with US $25 as compensation.

Participants were instructed to provide a saliva sample at four specific times daily: (1) in the morning immediately upon waking and before getting out of bed, (2) 30 minutes after waking, (3) before lunch, and (4) before bed. The 16 prelabeled salivette swabs were sent in tubes labeled and colored to identify the day of collection (2-5) and one of the above 4 general collection times. Participants were instructed to refrain from eating, drinking, or brushing their teeth before the collection of saliva samples. They were also instructed not to use any tobacco or caffeinated products 30 minutes before collection.

To collect a saliva sample, the participants opened the prelabeled tube and tipped the container to allow the swab to fall in their mouth, taking care to avoid touching the swab with their hands. They gently rolled the swab over their tongue until fully saturated. They then placed the swab back into the tube, avoiding touching the swab, and replaced the cap on the tube. The capped tube was placed inside the provided plastic bag and stored in the fridge (4 °C). After successful saliva collection, the participants shipped the salivettes, the completed collection sheet, and the medication use form in the preaddressed mailer to the MIDUS Biological Core at the Harlow Center for Biological Psychology at the University of Wisconsin–Madison. Samples were stored at –60 °C upon receipt. Salivettes were thawed and centrifuged at 3000 rpm for 5 minutes. Cortisol concentration was determined by chemiluminescence using a commercially available immunoassay (Tecan, IBL International) with intra-assay and inter-assay coefficient of variations below 5% [26]. Per participant, cortisol levels were assayed at each of the 16 sampling times. The participant was asked to write the specific time of day in the provided space on the salivette collection tube and on the provided saliva collection sheet. The participant was provided with an instructional insert and video demonstrating proper use of the salivettes, as well as access to a toll-free 1-800 number to reach research staff if additional instructions were needed.

### Measures

#### Measures Overview

Daily interviews included questions about experiences and events in the past 24 hours concerning time use, health behaviors, work productivity, positive and negative affect, physical symptoms, cognitive functioning, and daily stressors. Table 1 provides an overview of measures, including response options, additional operational detail, the wave each measure was first introduced, and example references. Below, we describe the main domains assessed in subcategories organized by the DISE, daily events, daily emotional experiences, daily physical health indices, daily cognitive health, and daily biomarkers. Full details of these questions are freely available [25].

**Table 1:** NSDE^a^ measures.

Measure					Response options		Items or operational details		Wave first introduced
**Daily Inventory of Stressful Events (DISE)**									
	**Stressor exposure [22]**			Yes/no		Composite across 6 stressor types		1	
		Stressor type [27]		Yes/no		Arguments, avoided arguments, work, home, discrimination, network, other		1	
		Who was involved [28]		Multiple options		Spouse or partner, child, parent, sibling, friend, coworker, etc		1	
		When did it happen [29]		Yesterday/today; AM/PM		Yesterday vs today; What time of day?		1	
		**Stressor appraisals**							
			Severity [30]	None at all – very		How stressful was this for you?		1	
			Control [31]	None at all – a lot		How much control did you have over the situation?		2	
			Resolution [32]	Yes/no		Is the situation resolved?		2	
			Resource risk [6]	None at all – a lot		Financial, health and safety, time schedule, socioemotional		1	
	Stressor reactivity [33]			Calculated slope		Emotional, behavioral, or biological reactions to stressors on the same day		1	
	Stressor residue [32]			Calculated slope		Prolonged responses extending to the following day		1	
	Stressor diversity [34]			Calculated index		Dispersion of stressors across multiple domains; Can be calculated using the Shannon entropy index		1	
	Anticipatory stress [35]			Not at all – very		How stressful do you expect tomorrow to be?		3	
**Daily events**									
	**Positive events [36]**			Yes/no		Interaction, work, home, network, other		2	
		Who was involved [36]		Multiple options		Spouse or partner, child, parent, sibling, friend, coworker, etc		2	
		When did it happen [36]		AM/PM		What time of day?		2	
		Anticipatory pleasantness [36]		Not at all – very		How pleasant do you expect tomorrow to be?		3	
	**Emotional support exchanges [37]**			Yes/no		Give or receive emotional support		1	
		Who was involved [37]		Multiple options		Spouse or partner, child, parent, sibling, friend, coworker, etc		1	
	Everyday discrimination [38]			Yes/no		9 items with follow-up reasons for discrimination (eg, race, gender, and age)		2	
	**Time use**								
		Work, volunteer, assistance [39]		Hours/minutes		How much time spent working, volunteering, and providing unpaid assistance		1	
		Physical activity, leisure, television [40]		Hours/minutes		How much time spent on physical activity, leisure, and television		1	
**Daily emotional experiences**									
	Negative affect [41]			None of the time – all of the time		14 items for “How much of the time today did you feel...[affect item]”		1^b^	
	Positive affect [41]			None of the time – all of the time		13 items for “How much of the time today did you feel...[affect item]”		2	
**Daily physical health indices**									
	**Health behaviors**								
		Cigarette smoking [42]		Quantity		How many cigarettes did you smoke?		1	
		Vape or e-cigarettes		Hours/minutes		How much time spent vaping or using e-cigarettes		3	
		Alcohol drinks [43]		Quantity		How many drinks did you have?		1	
		Fast food		Yes/no		Did you eat from a fast-food restaurant?		3	
	**Self-rated physical symptoms**								
		Experience [44]		Yes/no		Headache, fatigue, fever, muscle weakness, chest pain, etc		1	
		Severity [44]		Very mild – very severe		How severe was this symptom?		2	
	Sleep quantity [45]			Hours/minutes		How much time did you spend sleeping?		1	
	Medications [46]			Yes/no		Over-the-counter or prescription allergy, inhaler, antidepressant, etc		2	
**Daily cognitive health**									
	**Memory lapses**								
		Frequency [47]		Yes/no		Forget errand or chore, medication, appointment, someone’s name		R	
		Irritation [47]		Not at all – very much		How much did forgetting these things bother you?		R	
		Interference [47]		Not at all – very much		How much did forgetting these things interfere with your routine?		R	
	Unconstructive repetitive thoughts [48]			None of the time – all of the time		Think about personal problems or concerns, trouble concentrating, etc		R	
**Daily biomarkers**									
	Cortisol [46]			Salivette sample		Samples drawn at wake, 30 minutes after wake, before lunch, and before bed. Can be calculated as area under the curve, cortisol awakening response, and diurnal range as examples.		2	
	Alpha amylase [30]			Salivette sample		Samples drawn at wake, 30 minutes after wake, before lunch, and before bed. Can be calculated as morning decline and diurnal rise slopes.		R	

^a^NSDE: National Study of Daily Experiences.

^b^Wave 1 included 6 items from the Nonspecific Psychological Distress Scale. Beginning in wave 2, additional negative affect items were added from the Positive and Negative Affect Schedule. Example publications for each measure are provided in the citation next to the measure’s construct.

#### Daily Inventory of Stressful Events

Daily stress processes are assessed using the DISE [6]. Participants are posed with stem questions to assess different types of stressors (ie, arguments, avoided arguments, work overloads, home overloads, network stressors, discrimination stressors, and other stressors). For each stressor endorsed as having occurred, participants are asked additional questions on the content classification of the stressor. Specifically, participants shared who was involved in the event (eg, spouse or partner, friend, and coworker) and when the event happened (yesterday vs today; AM/PM). Next, participants shared their appraisals of the stressor, including (1) how stressful the event was (ie, *severity*) on a scale from none at all to very, (2) how much control they had over the situation (ie, *control*) on a scale from none at all to a lot, (3) whether the situation was resolved (ie, *resolution*) with a yes or no response, and (4) the areas of life at risk due to the stressor (ie, *resource risk*: financial, health and safety, time schedule, and socioemotional). Investigators can link information from the DISE (eg, exposure to daily stressors) to other variables from the NSDE protocol (eg, negative affect) to calculate stressor reactivity (ie, emotional, behavioral, or biological reactions to stressors on the same day) and stressor residue (ie, prolonged responses extending to the following day known as residue) components of the stress process model. Investigators can also measure stressor diversity (ie, dispersion of stressors across multiple domains) by calculating an entropy index. Before ending each call, participants share their forecasting of anticipatory stress by sharing how stressful they expect tomorrow will be on a scale from not at all to very.

#### Daily Events

##### Positive Events

Daily positive events are measured with questions about the occurrence of positive experiences: positive interactions, positive experiences at work or in a volunteer position, positive experiences at home or within their social network, spending time in nature, and any other positive event [49]. For each event endorsed, participants are asked follow-up questions on who else was involved, when it happened, and additional emotional reactions. Before ending each call, participants are asked to forecast how pleasant they expect tomorrow will be on a scale from not at all to very.

##### Emotional Support Exchanges

Participants reported whether they gave or received emotional support and specified who was involved with these exchanges (eg, spouse or partner, child or grandchild, parent, sibling, friend, or coworker).

##### Everyday Discrimination

Daily discrimination is assessed using a measure adapted from Williams and colleagues’ [50] 9-item Perceived Discrimination Scale. The scale was designed to capture recent experiences that involve character assaults (eg, treated with less courtesy or respect than others, treated as if inferior or dishonest) that may lead to interference with one’s socioeconomic position. A follow-up question is asked pertaining to the reasons for the perceived discrimination (eg, race, gender, and age).

##### Time Use

Participants share how much time (in hours and minutes) they spent working, volunteering, providing unpaid assistance, in physical activity (lite, moderate, and vigorous), leisure activities, and watching television.

#### Daily Emotional Experiences

Daily positive and negative affect are measured using an adapted inventory of emotions from the Nonspecific Psychological Distress Scale developed for the original MIDUS survey coupled with questions from the Positive and Negative Affect Schedule calibrated for daily affect [51]. Each day, respondents indicate how frequently they felt each of 14 negative emotional experiences (eg, feeling so sad that nothing could cheer them up, that everything was an effort, hopeless, and lonely) and 13 positive emotional experiences (eg, enthusiastic, attentive, and proud) over the past 24 hours on a 5-point scale ranging from “none of the time” to “all of the time.” The positive and negative affect measures have shown good internal consistency across both samples (core and refresher; Cronbach α in the high 0.8s and low 0.9s) and adequate between- and within-person reliabilities [52].

#### Daily Physical Health Indices

##### Health Behaviors

Each day, participants report engaging in (yes/no) and, if so, the number of cigarettes smoked, time spent vaping or using e-cigarettes, number of alcohol drinks they consumed, and whether they ate a meal from a fast-food restaurant.

##### Self-Rated Physical Symptoms

Daily physical symptoms are assessed using an adapted version of Larsen and Kasimatis’ [53] symptom checklist. We omitted items that overlapped with the psychological distress scale (eg, “urge to cry”). The checklist asks about the occurrence and severity of 19 symptoms, including aches (headaches, backaches, and muscle soreness), gastrointestinal symptoms (poor appetite, nausea or upset stomach, constipation or diarrhea), upper respiratory symptoms (sore throat, runny nose), and menopausal symptoms (hot flashes or flushes), as well as an open-ended item. Each day, the respondents indicate if they experienced each symptom and the degree of severity on a 10-point scale from very mild to very severe. These questions predict chronic illness incidence and increased likelihood of functional limitations 10 years later [44].

##### Sleep Quantity

Each day, participants report the time (in hours and minutes) they spent sleeping the previous day or night.

##### Daily Medications

Participants reported “yes/no” to taking the following medications: over-the-counter or prescription allergy medications, steroid inhalers, oral steroids, creams or ointments containing cortisone, birth control pills, other hormonal medications, antidepressant or antianxiety medications, steroid nasal sprays, and corticosteroid injections.

#### Daily Cognitive Health

##### Memory Lapses

The Daily Memory Lapses Checklist [47] measures the frequency, irritation, and interference of memory lapses in daily life. *Frequency* was captured by respondents indicating (yes/no) whether they encountered the following prospective memory lapses (forgetting an errand or chore, medication, to finish something you started, an appointment, why you entered a room, someone’s name, a word, or important information) and retrospective memory lapses (someone’s name, where you put something, where something was placed, a word during a conversation, or something you wanted to remember). As a measure of *irritation*, respondents answered “How much did forgetting these things bother you?” on a scale from not at all to very much. As a measure of *interference*, respondents answered “How much did forgetting these things interfere with your routine?” on a scale from not at all to very much.

##### Unconstructive Repetitive Thoughts

Six items on unconstructive repetitive thoughts assessed cognitive interference each day [54]. On a scale from none of the time to all of the time, respondents shared how often they had trouble concentrating, thought about personal problems or concerns, experienced thoughts that were difficult to stop, had thoughts keep jumping into their head, thought about situations that upset them, and thought about their financial situation.

#### Daily Biomarkers

##### Cortisol

Saliva was self-collected 4 times per day for 4 consecutive days. Cortisol levels are used to determine cortisol awakening response and area under the curve. The area under the curve was determined using the raw cortisol values of each of the 16 samples compared to ground [55]. Cortisol awakening response was determined using methods described in [46] (see also [56]). These measurements are critical to assessing a blunted cortisol response, indicative of chronic stress [57]. Compliance to the saliva collection protocol was previously validated [46,58-60].

##### Alpha Amylase

In addition to cortisol metrics computed from the saliva samples, raw and weight metrics of alpha amylase are computed and available for each of the 16 sampling times (ie, 4 times per day—at wake, 30 minutes after wake, before lunch, and before bed) for 4 consecutive days.

#### Analytic Approaches and Considerations

The NSDE presents a wide range of analytic opportunities when working with its measurement burst design of microlongitudinal daily diary assessments nested within macrolongitudinal assessment waves repeated every 9-10 years. While comprehensive coverage of all analytic approaches and considerations relevant for NSDE data is outside the scope of this protocol paper, we have specified below 3 approaches that characterize past work and guide future inquiries using NSDE data.

#### Opportunities for Descriptive Analysis and Aggregating Across Days of Assessment

Eight days of information provide robust metrics of a person’s experiences and health beyond a single snapshot of time. Repeated measurements of time-varying constructs (ie, daily assessments of stress, affect, physical symptoms, etc) create analytic opportunities to decompose variation at between-person (eg, individual differences) and within-person (eg, day-level) levels. As one example, Scott and colleagues [52] used NSDE data as part of a coordinated analysis of intensive repeated measurement studies that descriptively demonstrated significant between-person variation, within-person variation, and reliability of daily negative affect and daily positive affect. This kind of descriptive analysis provides statistical justification for research questions and hypothesis development at multiple levels of analysis when using data sources like the NSDE.

Researchers can also aggregate values of daily experiences and health outcomes across the 8 days of assessments to create person-means that can be used to examine individual differences in person-level health outcomes. For example, Charles et al [61] averaged across study days in the NSDE 2 and NSDE R1 to create two groups of participants (a group that reported stressors vs a group that did not report any stressors across the 8 days of assessment). This between-person group variable (no stressor group vs stressor group) was then used as a predictor of person-level physical and cognitive health variables in linear regression models. This work and others document how daily assessments can be aggregated to create a person mean index relevant to study individual differences in health and well-being outcomes across the adult life span.

#### Analysis Focusing on Daily Dynamics Within Persons Over Time

Multilevel modeling (MLM) can flexibly manage the nested structure of NSDE data to evaluate daily dynamics within people over time [62]. Three-level MLMs can be used to model time-varying constructs (eg, stressors, affect, and physical symptoms) across study days (level 1, day level; capturing microlevel daily variation) nested within waves of assessment (level 2, wave level; capturing macrolevel changes across years) nested within persons (level 3, person level; capturing individual differences). MLM can also incorporate cohort-level differences to test specific hypotheses about how historic periods (eg, the Great Recession and the COVID-19 pandemic) impact daily experiences and daily processes (eg, stressor reactivity) as well as how these phenomena change over time.

Almeida and colleagues [22] used 3-level MLMs to model wave-level changes in daily stress processes across 20 years of adulthood (NSDE 1, NSDE 2, and NSDE 3). Stressor reactivity (the difference in negative affect on days when a stressor was reported compared with days when a stressor was not reported) was modeled at level 1 (day level), where day-level negative affect was regressed on a day-level stressor exposure variable. Macrolongitudinal changes in stressor reactivity across 20 years were modeled at level 2 (wave level), and baseline age moderation of changes in stressor reactivity was modeled at level 3 (person level). Researchers continue to leverage the adult life span samples and multiple waves of NSDE data to model age differences in constructs like stressor reactivity [63] and developmental trajectories of constructs such as affect [41] and control [31].

The NSDE provides a pivotal resource for researchers to examine daily linkages among time-varying behaviors, experiences, appraisals, and health outcomes using MLM. As one example, Cichy et al [28] leveraged 2-level MLMs to examine race differences in daily associations between family support exchanges and daily well-being, and whether family support buffered or amplified stressor reactivity. The daily diary design provided the capacity to simultaneously examine individual differences (ie, race) in the within-person associations between family support exchanges and well-being [28].

#### Recent Advancements in Macrolongitudinal Change in Daily Dynamics

Most prior research has primarily used a 2-step approach to examine individual differences and patterns of change among short-term within-person associations, where within-person daily associations (eg, stress reactivity slopes) are first extracted from an MLM and then subsequently included as an individual difference variable in follow-up models. Recent advances can now simultaneously model individual differences in day-level constructs (eg, stress reactivity); how that construct (eg, stress reactivity) changes over time (wave-level); and whether individual differences in the rates of change account for changes in health and well-being outcomes (person-level), modeled as random slopes within a joint modeling multilevel structural equation modeling (MSEM) framework [33,64]. Modeling these effects simultaneously within a single statistical model allows for a more computationally efficient modeling of the variability within and across levels of analysis. The MSEM approach permits a sophisticated linking of processes operating on differing timescales, where short-term daily processes that unfold over time can simultaneously be treated as antecedent and outcomes of slower developing changes in health and well-being.

## Results

Since 1995, data collection has occurred every 9-10 years. The most recent data collection is ongoing through 2027. Since 1995, a total of 3510 adults, ranging in age from 24 to 97 years, have participated in the project. The NSDE has accumulated 42,243 daily interviews across multiple waves of data collection. All NSDE data are held within the online framework of the MIDUS study, with archived and continuously updated datasets made available to the research community [19].

## Discussion

### Impact of the NSDE

The NSDE is a foundational resource for understanding how the rhythms of daily life shape long-term psychological and physical health. This paper outlines the NSDE protocol and highlights its integration of daily diary methods, salivary biomarkers, and longitudinal health data spanning more than 20 years and involving over 3500 participants. By capturing the microlevel dynamics of stress, emotion, and social interaction—and linking them to long-term outcomes—the NSDE provides a uniquely rich and nuanced view of human development across adulthood. The study’s methodological innovations and scientific breadth make it a critical tool for identifying biopsychosocial pathways through which everyday experiences accumulate and contribute to disparities in health, well-being, and aging.

The NSDE data have transformed our understanding of well-being across the adult life span. NSDE has tested and inspired theoretical models (eg, Daily Stress Process Model [4]) about how daily experiences influence health and mortality. For example, studies testing the Daily Stress Process Model have revealed how stressors piling up over time (ie, stressor accumulation) and of greater severity are each related to both higher levels of distress [2,64-66], and worse physical health and functioning, including greater inflammation [67,68], higher allostatic load [69], and long-term detriments to physical and mental health [33,70-72]. Using NSDE, researchers have examined how the experience of stress affects the health and well-being of some people more than others, with differences occurring as a result of age [73], socioeconomic status [74,75], sexual orientation [76], discrimination [77], and exposure to early childhood adversity [78]. Using these data, researchers have examined how stress processes change over time [22] and how they are influenced by global events, such as the Great Recession [65,79].

The above provides a few examples of the many findings from studies using NSDE, but many opportunities remain. Below, we mention several of the most pressing research questions in the field of adult development that can be addressed with available NSDE data.

### Identifying Modifiable Factors Related to Cognitive Decline and Dementia

Age is the strongest risk factor for cognitive decline and for dementia. By 2060, almost a quarter of Americans will be over 65 years old, making the search for lifestyle factors that may slow cognitive decline or delay the onset of dementia a national priority. Researchers have used the NSDE in studies that document the importance of an active and varied lifestyle in predicting cognitive decline [17] and how different types of stressor exposure are related to cognitive function [80]. Yet, testable questions remain. For example, daily memory lapses, a predictor of later cognitive decline that varies by gender and education [47], are a relatively new addition to NSDE. Researchers can examine interrelationships between memory lapses and other daily activities, as well as combining information collected from other MIDUS studies to examine how they are related to physiological processes and cognitive functioning.

### Understanding How Everyday Thoughts and Behaviors Influence Gene Expression

Social genomics is a relatively new area of study that focuses on how our social environment affects gene expression and functioning [81]. The NSDE includes genome-wide RNA profiles related to inflammatory and antiviral activity, as well as gene methylation to assess the pace of biological aging (epigenetic age acceleration) and epigenetic mortality risk for over 1000 people. By linking these genetic differences with daily social processes, researchers can discover the genetic mechanisms through which these daily processes are linked to cognitive, physical, and psychological well-being.

### Midlife Predictors of Health in Older Adulthood

Longitudinal data have provided important insight into how earlier life experiences influence later health [82]. Most longitudinal studies, however, consist of one-time surveys that provide overall ratings that are subject to memory bias [83]. In contrast, NSDE provides insight into daily lives as they are lived and uses data from multiple days to predict health, functioning, and longevity years later. Understanding, for example, how simple daily behaviors decades earlier predict cognitive decline or greater risk for disease will help identify behaviors that have lasting influences on our health and well-being.

### Changing Social and Historical Effects

In addition to studying how individual differences influence health trajectories, researchers can also examine how larger societal changes influence health and well-being. In the past several decades, for example, a greater percentage of adult children live with their parents, and a growing number of men and women are living alone [84]. NSDE examines different cohorts of adults throughout the first two decades of the twenty-first century, providing insight into how changes in social structure shape daily life. In addition, the study has followed people before and after the Great Recession. Researchers have documented historical changes in physical health and well-being [85] and how historical time periods have a greater impact on some age groups [65] that may influence people’s attitudes of aging [86]. Yet, we have more to learn regarding how societal changes influence individual lives.

### Data Collection Considerations and Lessons Learned From the NSDE

Any large-scale data collection presents challenges that can impact participant participation, compliance, and data quality. Standardizing collection protocols is essential when implementing naturalistic assessments of daily experiences and collecting saliva samples in people’s daily surroundings (ie, ecological momentary assessment rather than a laboratory setting).

Kits with clearly labeled contents and instructions help reduce participant burden and increase compliance. Included inserts contain colorful instruction sheets with diagrams and scannable QR codes for instructional videos prepared by NSDE staff. The fillable collection sheets allowed the participants to record the day and time of salivary sample collection as well as any noted issues they encounter with that collection point (ie, dropped swab, missed timepoint, etc). Participants recorded all medications and supplements on a medication use form, allowing researchers to analyze potential correlations between medication use and measured cortisol levels. To maximize salivary cortisol reliability and reproducibility, the kit instructions emphasized proper storage of salivettes. Completed saliva sample shipments were tracked to ensure timely delivery and processing.

Recruitment can be difficult in a study requiring daily commitment across multiple waves of longitudinal follow-up. For initial recruitment into the NSDE, highly trained interview staff clearly communicated the study purpose and used effective strategies to reach potential participants. Participants also had access to staff for questions about the study procedures using a toll-free 1-800 number. Additionally, standardization of the telephone interview script, questions, and response choices allowed for reliable data collection across participants and cohorts. This is consistent with research that highlights the integral nature of personal engagement to increase recruitment, adherence, and retention [24].

### Statistical Considerations When Using NSDE Data

With 8 days of daily diary data nested within up to 3 waves of assessment across 20 years of adulthood, analysts of NSDE data should be aware of potential design limitations when conducting their statistical analysis. Below, we briefly discuss statistical considerations when dealing with attrition across assessment waves and handling missing data in the NSDE and point the reader to didactic overviews of MLM [87] and understanding threats to validity of longitudinal designs. For example, MacDonald and Stawski [88] provide guidelines for handling attrition, retest effects, and missingness in longitudinal designs like the NSDE.

Participant attrition can be due to a multitude of factors, including busyness (no time), illness, lack of interest, relocation, or death. Participants may not complete the entire study or selectively complete certain components of the protocol. Researchers can test whether the attrition or selective missingness of certain assessments is nonrandom and the extent to which rates of change may be influenced or biased by this threat. Assessing reasons and characteristics associated with attrition as well as incorporating parameters for attrition into statistical models can improve the conclusions drawn from the analyses, inform the generalizability of the analytic sample, and isolate possible influence from attrition processes [88-90].

Missingness across days and waves of assessment can influence study results in various ways, ranging from reducing statistical power to potentially biasing parameter estimates [89]. Modern statistical approaches (eg, likelihood-based estimation and multiple imputations) can be implemented to obtain unbiased model estimates when working with missing data. As one example, full information maximum likelihood (FIML) estimation yields unbiased parameter estimates under conditions where data are assumed to be missing at random [91,92] and are based on all available information (in contrast to listwise deletion or imputed data points, FIML uses all available data and preferentially weights cases with more observations). Likelihood-based approaches such as FIML are most appropriately used on larger sample sizes [93], making the NSDE an ideal dataset for its use.

### Conclusions

Daily diary studies capture the thoughts and behaviors that constitute the fabric of daily life. NSDE is an example of a daily diary study whose measurement burst design complements the wide assessment spacing of a macrolongitudinal study. In this manner, NSDE is ideal for identifying processes and capturing experiences that would otherwise be missed if just viewing health and aging from the wider assessment windows. One aim of this paper was to provide information for people interested in using the NSDE methodology for novel studies. Applying the NSDE protocol to new samples would provide a greater understanding of how daily life impacts health across different populations. A second aim was to provide information for researchers interested in using existing NSDE data. We suggested several quantitative models and future research directions for researchers interested in using NSDE to expand our knowledge of how to live healthier, longer, and more fulfilling lives. Cumulatively, these theoretical and empirical contributions highlight avenues for innovation, intervention, and application [4] and serve as examples for which research using NSDE, or applying the NSDE protocol in new studies, can continue to inform our understanding of influences of health from a microlongitudinal perspective.

## Abbreviations

DISE: Daily Inventory of Stressful Events
FIML: full information maximum likelihood
MIDUS: Midlife in the United States
MLM: multilevel modeling
MSEM: multilevel structural equation modeling
NSDE: National Study of Daily Experiences

## Data Availability

The datasets generated during this study are available at [19].
